# Gut microbiome reflect adaptation of earthworms to cave and surface environments

**DOI:** 10.1186/s42523-022-00200-0

**Published:** 2022-08-05

**Authors:** Xin Gong, Ting-Wen Chen, Lili Zhang, Václav Pižl, Karel Tajovský, Miloslav Devetter

**Affiliations:** 1 Soil Ecology Lab, College of Resources and Environmental Sciences, Nanjing Agricultural University, Nanjing, 210095 China; 2 Institute of Soil Biology, Biology Centre, Czech Academy of Sciences, Na Sádkách 7, 37005 České Budějovice, Czech Republic

**Keywords:** Cave, Earthworms, Functional diversity, Gut microbiome, *Rrn*, Network stability

## Abstract

**Background:**

Caves are special natural laboratories for most biota and the cave communities are unique. Establishing population in cave is accompanied with modifications in adaptability for most animals. To date, little is known about the survival mechanisms of soil animals in cave environments, albeit they play vital roles in most terrestrial ecosystems. Here, we investigated whether and how gut microbes would contribute to the adaptation of earthworms by comparing the gut microbiome of two earthworm species from the surface and caves.

**Results:**

Two dominant earthworm species inhabited caves, i.e., *Allolobophora chlorotica* and *Aporrectodea rosea*. Compared with the counterparts on the surface, *A. rosea* significantly decreased population in the cave, while *A. chlorotica* didn’t change. Microbial taxonomic and phylogenetic diversities between the earthworm gut and soil environment were asynchronic with functional diversity, with functional gene diversity been always higher in earthworm gut than in soil, but species richness and phylogenetic diversity lower. In addition, earthworm gut microbiome were characterized by higher *rrn* operon numbers and lower network complexity than soil microbiota.

**Conclusions:**

Different fitness of the two earthworm species in cave is likely to coincide with gut microbiota, suggesting interactions between host and gut microbiome are essential for soil animals in adapting to new environments. The functional gene diversity provided by gut microbiome is more important than taxonomic or phylogenetic diversity in regulating host adaptability. A stable and high-efficient gut microbiome, including microbiota and metabolism genes, encoded potential functions required by the animal hosts during the processes of adapting to and establishing in the cave environments. Our study also demonstrates how the applications of microbial functional traits analysis may advance our understanding of animal-microbe interactions that may aid animals to survive in extreme ecosystems.

**Supplementary Information:**

The online version contains supplementary material available at 10.1186/s42523-022-00200-0.

## Background

Terrestrial caves differ from surface habitats and are regarded as “natural laboratories” [1]. Organisms in caves are subjected to strong selective pressures that are rather different from surface, such as constant dark and static climatic conditions [2]. Further, food resources for animals are depleted in cave as compared to the surface, which rely on photosynthesis for primary production [3]. Instead, food webs in caves are based on microbes, making the interactions in soil food webs in cave hitherto unknown [4]. Cave-dwelling animals therefore have to modify their feeding strategies in ways different from surface animals to cope with food deficiency [5].

On the surface, earthworms are common ecosystem engineers which provide a variety of ecosystem functions and services [6]. Some of them are able to establish stable populations in subterrain caves [7, 8]. Species that survive in caves need to adjust to the rather different environment compared with those living in other ecosystems. Compared to cave arthropods that are usually pale and blind [9], cave earthworms appear to be similar in external morphological characters to their relatives inhabiting surface layers. However, adaptive strategies of earthworms to cave environments and the manner how different earthworm species sustain populations in the resource-limited environments remain obscure.

Gut-associated microbes are considered as “nutrient factories” that increase host fitness in many ways [10–12]. With the help of diverse functional genes encoded by gut microbes, the hosts may digest a wide range of compounds, thereby surviving in unfavorable environments [13]. Reciprocally, gut microbiome are regulated by the physiological conditions and feeding diets of the hosts [14, 15]. For example, the community structure and functional potential of the gut microbiome differ in gut compartments and are associated with the feeding strategies of the hosts [16]. Besides, species-specific effects from the hosts on gut microbiome have also been discovered for soil animals [17]. Therefore, cave earthworms are presumably to harbor specific gut microbiome which may help them to adapt to the cave environment, and the same earthworm species may reshape their gut microbiome when living in a different environment.

Here, we asked whether gut microbiome of cave earthworms differ from the respective earthworm species inhabiting surface and if the gut microbiome may provide functions for earthworm adaptation to the optimal environments (i.e., surface or cave). We explored patterns of gut bacterial communities and predicted functional genes encoded by gut bacteria as well as the copy numbers of the 16S rRNA gene to reflect the ecological strategies of bacterial communities in nutrient exploitation [18]. We also examined networks of gut bacterial communities which suggest interactions and stability of the gut microbiome [19]. We hypothesized that 1) gut bacterial communities differ between cave and surface earthworm populations in taxonomic, functional and phylogenetic diversity and the difference depends on the species adaptability to environments; 2) The assembly processes of gut microbiome are more deterministic than that of soil microbiota since cave earthworms are likely to select bacteria of certain functions for the adaptation in the specific environment; 3) Gut microbiome are characterized by less fast growing species and more stable networks when the earthworm hosts inhabit favorable environments.

## Methods

### Study sites and sampling

**Fig. 1:**
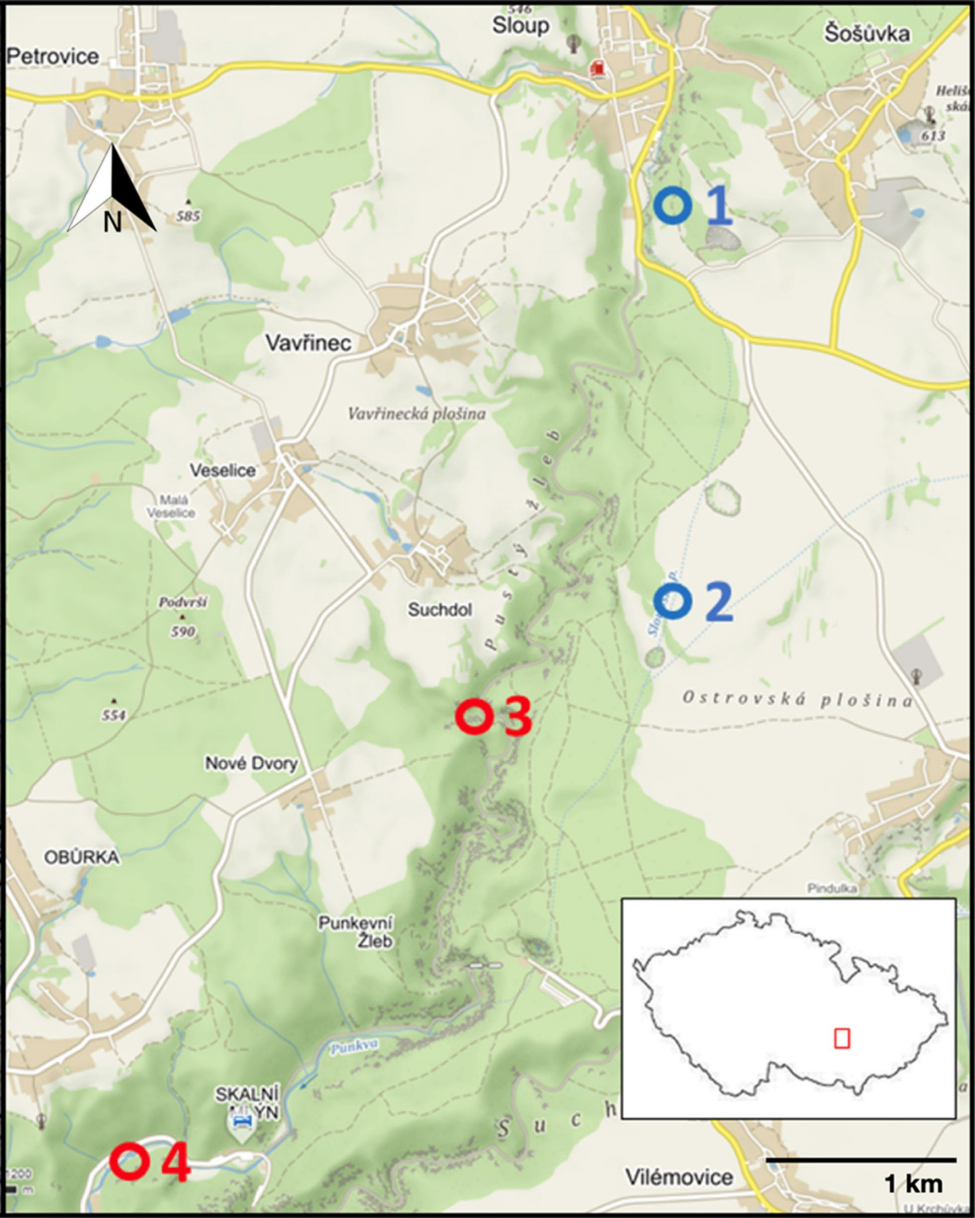
Location of sampling sites. Soil samples were collected from both cave (sites 1 and 2 corresponding to the Sloupsko-Šošůvské Caves and Amatérská Cave, respectively) and surface (sites 3 and 4). The earthworm *A. chlorotica* was sampled in sites 1 and 4, while *A. rosea* was sampled in sites 2 and 3

The study sites are located in two interconnected cave systems, the Amatérská Cave and the Sloupsko-Šošůvské Caves, in the Moravian Karst Protected Landscape Area in the south-east part of the Czech Republic (Fig. 1 and Table 1). The cave system is associated with streams, Sloupský potok and Bílá voda, which later merge to the Punkva River. The water mainly flows underground, dropping to the lime bedrock massif. The gallery-like caves were originally formed by streams and have been connected with the surface layers via water flow. Nowadays they are situated in the vadose zone far from the streams, with the only water supply being infiltration of rainwater through soil and bedrock rifts, except for extreme flooding events. The two sampled cave systems are separated by a series of water siphons and are accessible only through an artificial corridor. No bats are present here and support of organic matter is possible via floods only. Just the corridors of the Sloupsko-Šošůvské Caves (Site 1; Fig. 1 and Table 1) are connected directly with the surface. The sampling took place in the center of the caves, which were far away from the water siphons. Soils in all sites are of grey rendzina type, affected by close permanent or semi-permanent water stream. Vegetation of surface sites is very similar, represented by *Stellario nemorum*-*Alnetum glutinosae*. Soil pH values of the cave and soil substrates ranged between 8.5 and 7.0, soil organic carbon (SOC) 7–17.1 g/kg. [20].

**Table 1:** Abundance of earthworms (*Allolobophora chlorotica* and *Apporrectodea rosea*) and the soil properties in caves and surfaces. Values are presented as mean ± SD

Habitat		Cave	Surface	
Site^┼^	1	2	3	4
Longitude (°E)	16.74	16.74	16.72	16.7
Latitude (°N)	49.41	49.39	49.38	49.36
Earthworm identity	*A. chlorotica*	*A. rosea*	*A. rosea*	*A. chlorotica*
Earthworm abundance (ind. m^−2^)	20.7 ± 39.5	2.6 ± 1.1	71.7 ± 24.1	39.3 ± 18.4
pH	7.8 ± 0.1	7.9 ± 0.1	7.7 ± 0.1	7.8 ± 0.0
Conductivity (µS cm^−1^)	82 ± 15	107 ± 47	178 ± 32	242 ± 27
Soil HWC (mg kg^−1^)	461.7 ± 134.9	530.8 ± 400.4	93 ± 14.1	98.4 ± 20.3
Soil NH + 4 (mg kg^−1^)	4 ± 1.2	6.2 ± 6.4	1.6 ± 1.2	1.6 ± 0.3
Soil NO- 3 (mg kg^−1^)	54.4 ± 24.7	213.5 ± 294.2	16 ± 4.0	14.6 ± 9.8
Soil PO3- 4 (mg kg^−1^)	8.2 ± 3.6	9.9 ± 4.0	6.5 ± 1.4	8.2 ± 4.3
Soil SO2- 4 (mg kg^−1^)	33.9 ± 10.2	68.8 ± 60.5	14.9 ± 5.6	4.3 ± 0.5
Soil Cl^−^ (mg kg^−1^)	10.1 ± 3.3	14.1 ± 6.7	12.2 ± 7.3	2.6 ± 0.3
Soil Na^+^ (mg kg^−1^)	16.5 ± 5.5	23.9 ± 16.0	4.9 ± 1.7	1.6 ± 0.2
Soil K^+^ (mg kg^−1^)	15.8 ± 0.8	20.2 ± 7.7	14.9 ± 5.5	11.1 ± 3.9
Soil Mg^2+^ (mg kg^−1^)	12.8 ± 1.6	18.4 ± 12.4	10.6 ± 0.2	9.5 ± 0.0
Soil Ca^2+^ (mg kg^−1^)	149.7 ± 39.3	232.9 ± 77.6	104.2 ± 12.1	90.3 ± 18.4

^┼^Locations of sampling sites as shown in Fig. 1

Earthworms and soils from each site were sampled during the data 3–10 May 2016, which was spring for the sampling sites and corresponding to the growing season for the earthworms, evidenced by the casts they produced in the field. The sampling sites were surveyed for common soil animals, e.g., mesofauna springtails and macrofauna earthworms, and the present study was focused on the earworms. In the cave, the soil was mixed with earthworm casts (Fig. 1A), thus the sampling of cave soil was a mixture of soil and earthworm casts. On the surface, the soil sampling was conducted in an area dominated by a specific earthworm species. When sampling, five 5 × 5 m quadrats were randomly set up at each site with a distance of at least 10 m (Fig. 1B, C), and eight soil cores were collected and mixed for one composite sample for each quadrat. The soil cores were taken with a 2.5 cm diameter cylinder, and to a depth of 10 cm, or until reaching the rock. This method enabled at least 100 g of soil for each core. After soils were collected, the earthworms were dug out from each quadrat. In each quadrat, earthworms were hand-sorted and preserved in their living soils and were transported with ice to the lab. In the lab, the earthworms were identified to species level and stored in absolute ethanol prior to molecular gut content analysis. The abundance of the earthworms per site was calculated as the mean of earthworm density in the eight quadrats. After sampling, soils with earthworms submerged in their living soils were transported with ice to the lab. In the lab, the earthworms were fixed and stored in absolute ethanol and then identified to species level prior to molecular gut content analysis. Only two earthworm species were found in the cave systems, i.e., *Allolobophora chlorotica* and *Aporrectodea rosea* in Sloupsko-Šošůvské Cave (Fig. 1, site1) and Amatérská Cave (Fig. 1, site 2), respectively. *A. chlorotica* and *A. rosea* belong to the same family, i.e., Lumbricidae, and are both widespread in Europe [21].

### Molecular gut content analysis

Five individuals of earthworms from each site were dissected and separately used for DNA extraction during the molecular analysis. The earthworms were dissected aseptically under a stereomicroscope. An incision was made longitudinally along the body wall and the whole gut, from the clitellum to anus, was removed and placed in a 1.5 mL Eppendorf tube. Thereafter, total DNA of the gut content as well as soils were extracted using the FastDNA Spin Kit for Soil and the FastPrep Instrument (MP Biomedicals, Santa Ana, CA, USA). All steps were carried out following the manufacturer's instructions. The quality and quantity of the extracted DNA were certified with 1% agarose gel electrophoresis and a Nanodrop-2000 spectrophotometer (NanoDrop Technologies Inc. Wilmington, DE, USA), respectively. The V4 hypervariable region of the bacterial 16S rRNA gene was amplified and sequenced with a Miseq sequencer at the University of Illinois—Chicago with primers of 16S rRNA gene V4 region (FWD: 5’-GTGYCAGCMGCCGCGGTAA-3’; REV: 5’-GGACTACNVGGGTWTCTAAT-3’) were used following the EMP protocol (https://earthmicrobiome.org/protocols-and-standards/16s/). Negative controls that replace DNA templates with sterilized water were included in the amplification period. The raw sequences were deposited in NCBI Sequence Read Archive under the accession number PRJNA400302.

### Sequence data processing

Paired-end sequence data were joined, demultiplexed and analyzed using the QIIME 1.9.1 pipeline [22]. Briefly, sequence lengths < 200 bp, of average quality score < 20 or with ambiguous characters were discarded. After chimeras and singletons were removed, closed reference operational taxonomic units (OTUs) were clustered on the basis of 97% similarity. Taxonomy of bacterial OTUs was assigned using Greengenes v13_8. A phylogenetic tree was generated using “make_phylogeny.py” by the default setting of the “FastTree” method. The resulting OTU table was then rarefied to 9800 sequences per sample before further analysis.

### Statistical analysis

The 16S rRNA gene copy numbers as well as functional gene abundance were calibrated and predicted by PICRUSt [23]. The abundance of functional genes was predicted using a script predict_metagenomes.py implemented in the PICRUSt according to the recommended protocol. The predicted genes were then grouped at the first KEGG level using the script categorize_by_function.py implemented in the PICRUSt. Other statistical analyses were performed in R 4.0.0 [24]. Student’s t-test was used for comparing the mean abundance of earthworms between cave and surface. The standardized effect size of abundance weighted mean phylogenetic distance of the bacterial community was quantified using the function *ses.mpd* implemented in the R package “picante” [25]. OTU numbers, diversities and phylogenetic relatedness of microbial communities were compared between treatments using ANOVA followed by Tukey's HSD test. Community weighted means (CWM) of the 16S rRNA gene copy numbers were calculated with the equation: $\mathrm{CWM operon}={\sum }_{1}^{n}Pi*mi$, where *Pi* and *mi* is the proportion and operon numbers of each bacterial OTU. Pairwise correlations of the bacterial OTUs within treatments were calculated using the command sparcc with 1000 bootstraps in the program mother v.1.35.0 [26]. Significant correlations were set by R^2^ > 0.7 and *P* < 0.01. Topological properties of the bacterial community network of each treatment included (I) numbers of nodes and edges, (II) average degree, which measures network complexity, and (III) average path length (i.e., distance between any two nodes). Network properties were calculated using the “igraph” package in R [27].

## Results

### Earthworm abundance

The density of *A. chlorotica* in the cave and surface were 20.7 ± 39.5 (individuals/m^2^; mean ± SD) and 39.3 ± 18.4, respectively (Table 1). The density of *A. rosea* (2.6 ± 1.1) in the cave was much lower than surface (71.7 ± 24.1), with the differences were significant (t = 6.40, df = 8, *P* < 0.05).

### Microbial diversity

**Fig. 2:**
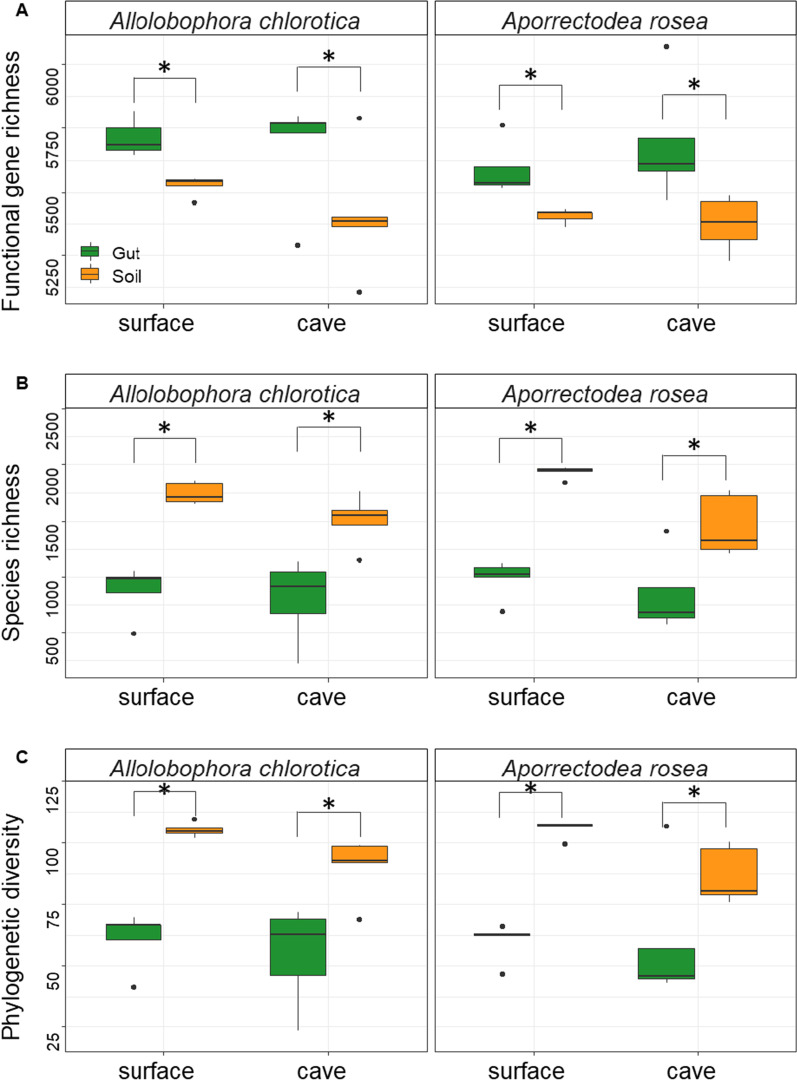
Functional (**A**), taxonomic (**B**) and phylogenetic (**C**) diversities of bacterial communities in the soil and gut of earthworms present in caves and the surface

Functional diversity inferred by the predicted functional gene richness was greater in the gut of earthworms than those in the soils (Fig. 2A). Gut bacteria of both *A. chlorotica* and *A. rosea* were functionally more diverse in the caves than in the surface (Fig. 2A). Regarding the eight categories of predicted functions, both *A. chlorotica* and *A. rosea* exhibited greater genes related to metabolisms (Additional file 1: Figure S1). Gut microbiome holds more abundance of functional genes than that of soils, but the difference of genes between cave and surface was not significant (Fig. 2A). Taxonomic and phylogenetic diversities of bacterial communities, however, were lower in the gut of earthworms than those in the soils (Fig. 2B and C). Regardless of gut or soil microbiota, the taxonomic and phylogenetic diversity was lower in the caves than in the surface for both *A. chlorotica* and *A. rosea* (Fig. 2B and C), with the greater reductions for *A. rosea* (F = 4.93 and 14.77 for *A. chlorotica* and *A. rosea*, respectively; *P* < 0.05).

### Phylogenetic relatedness

**Fig. 3:**
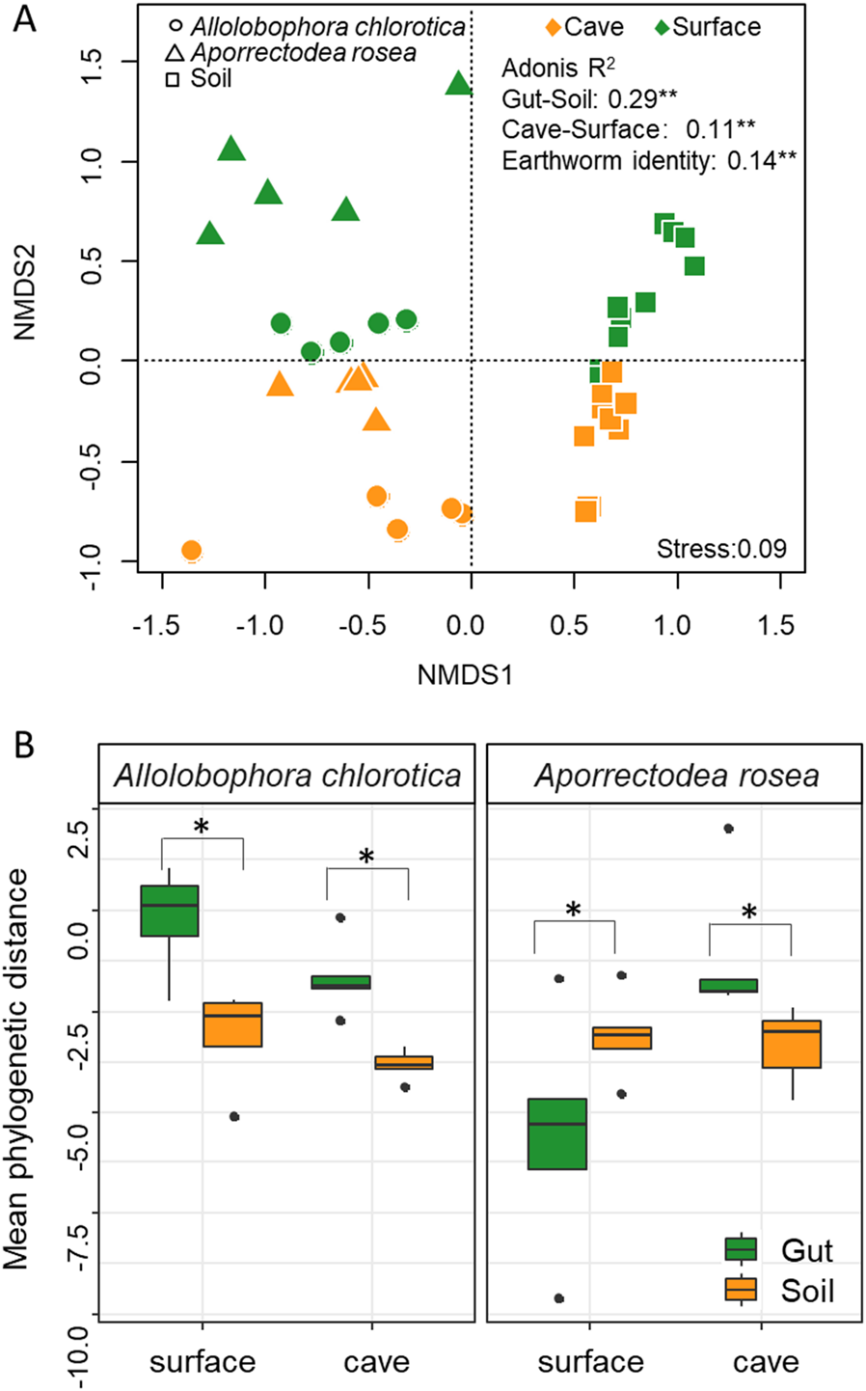
Ordination plot of microbiota (**A**) and the standardized effect size of mean phylogenetic distance of bacterial communities (**B**) in the soil and gut of earthworms present in caves and the surface

Except for the bacterial communities in the gut of *A. chlorotica* from the surface, which exhibited a random pattern of phylogenetic relatedness, the bacterial communities of all the other treatments showed phylogenetic clustering (Fig. 3). The standardized effect size of mean phylogenetic distance of soil bacteria was significantly lower than in the gut of *A. chlorotica* irrespective of the habitats (i.e., surface or cave; *P* < 0.05). However, in the gut of *A. rosea* bacterial communities were more phylogenetic clustered than the soil bacterial communities if they were collected from the surface but not the cave. For *A. chlorotica*, the mean phylogenetic distance of the gut bacterial communities was greater in the surface than cave, while for *A. rosea*, the reverse was true (Fig. 3B).

### Community weighted mean operon numbers

**Fig. 4:**
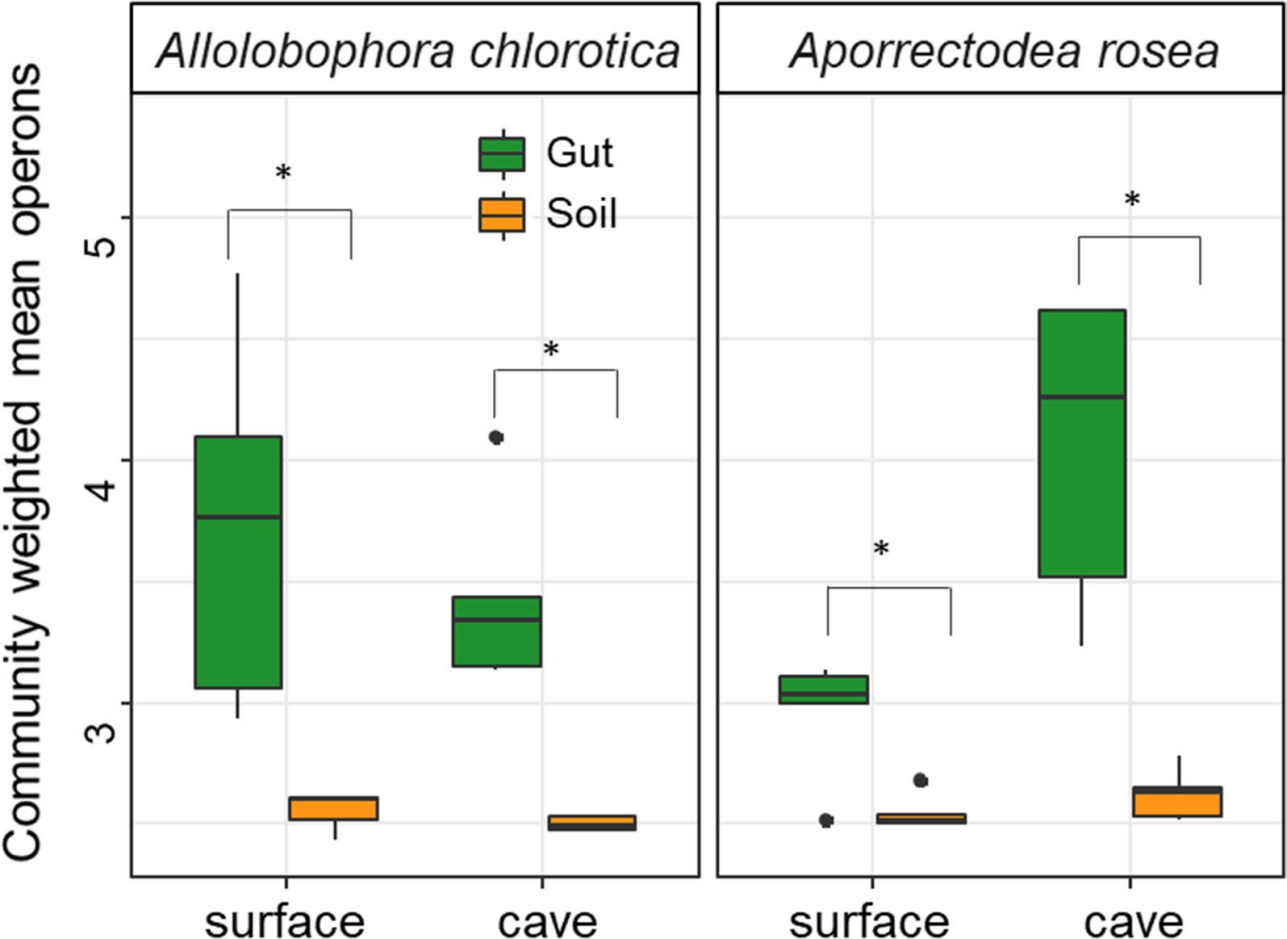
Community weighted mean (CWM) of 16S rRNA gene copy numbers in the soil and gut of earthworms present in caves and the surface. Copy numbers were estimated using PICRUSt and weighted values were obtained by multiplying copy numbers by the relative abundance for each operational taxonomic unit and taking the sum of these values for each community

For both earthworm species in general, the community-weighted mean of the 16S rRNA gene (*rrn*) copy numbers of bacteria were significantly higher in the gut of earthworms than that in the soils (mean value 3.8 and 2.5, respectively; Fig. 4). The CWM operon numbers were not different between the surface and cave soils (*P* > 0.05). However, in the gut of *A. chlorotica*, the value was significantly greater in the surface than in the cave, while an opposite pattern was found in the gut of *A. rosea* (*P* < 0.05).

### Co-occurrence networks of microbial communities

**Fig. 5:**
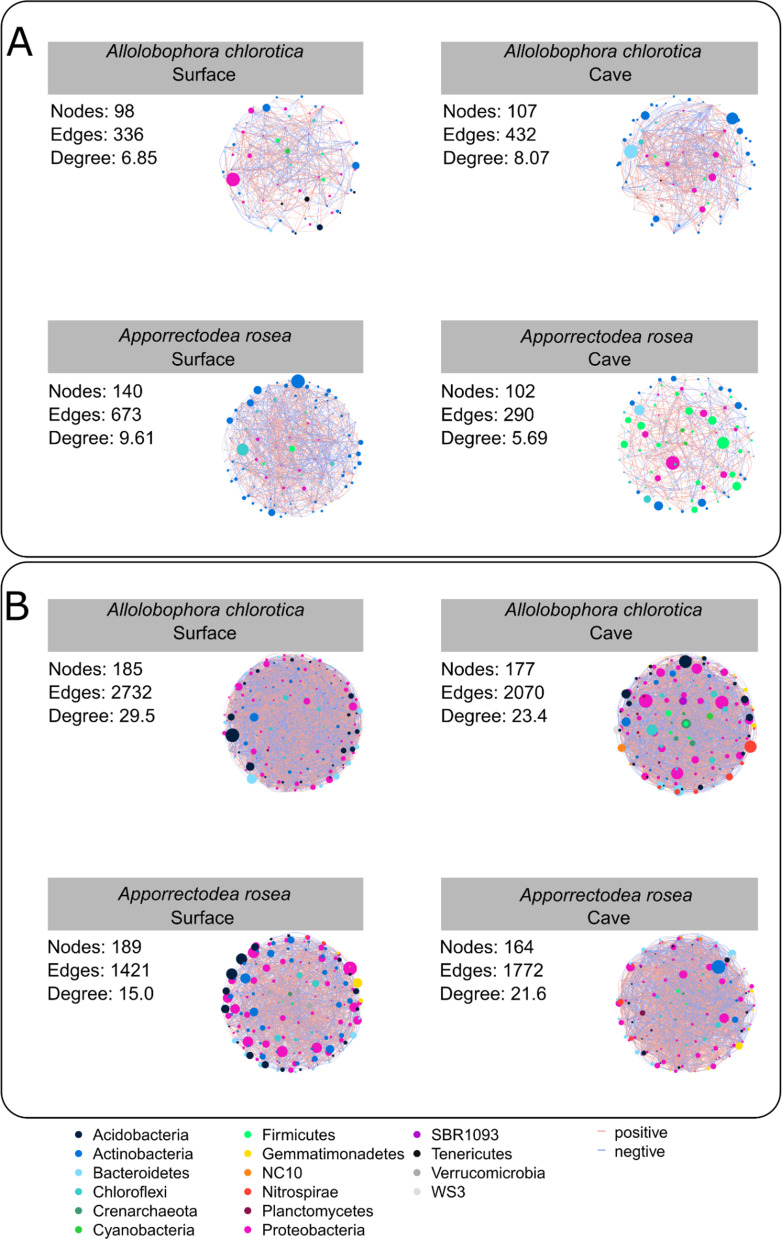
Co-occurrence networks of bacterial communities in the gut of earthworms (**A**) and soil (**B**) present in caves and the surface. Connections represent strong (R^2^ ≥ 0.7) and significant (P ≤ 0.01) correlations inferred by SparCC. The size of each node is proportional to the betweenness. Blue lines represent significant negative and red lines significant positive correlations. Node colors represent the OTU phyla

Networks mainly consisted of the most abundant phyla and comprised highly connected OTUs structuring densely connected groups of nodes (Fig. 5 and Table 2). The networks of bacterial communities in the gut of both earthworm species exhibited fewer degrees. The degree of the network in the gut of *A. rosea* was reduced ~ 50%, while *A. chlorotica* increased 18% when living in the cave as compared to the surface layers.

**Table 2:** Network topologies of the gut and soil microbiota

	*Aporrectodea rosea*				*Allolobophora chlorotica*			
	Surface (Site 3)		Cave (Site2)		Surface (Site 4)		Cave (Site 1)	
	Gut	Soil	Gut	Soil	Gut	Soil	Gut	Soil
Modularity	0.36	0.35	0.51	0.26	0.45	0.20	0.38	0.24
Nodes	140	189	102	164	98	185	107	177
Edges	673	1421	290	1772	336	2732	432	2070
Degree	9.6	15.0	5.6	21.6	6.85	29.5	8.07	23.3
Path length	3.1	2.7	3.56	2.4	3.1	2.33	3.3	2.5
Connectance	0.07	0.08	0.06	0.13	0.07	0.16	0.08	0.13
Diameter	9	6	8	7	8	7	8	7
Positive edges (P)	329	745	169	948	189	1430	229	1038
Negative edges (N)	344	676	121	824	147	1302	203	1032
P/N	0.95	1.10	1.39	1.15	1.28	1.09	1.12	1.01

## Discussion

*A. chlorotica* and *A. rosea* exhibited different variations of abundance in caves. Presumably, they might consume more forms of food with the aid of their gut microbes. Adaptation of animals to different environments usually requires physiological adjustments, including changes in biochemical activities. However, physiological or genetic-based adaptation of the earthworms usually takes generations, longer than the lifespan of individual earthworms. The gut microbiome, as part of the holobionts, may facilitate the animal hosts to adapt to unfavorable environments by a diverse encoding of genes [28, 29]. The gut microbiome of earthworms, therefore, could instantly be recruited by the host and help them to utilize more diverse food compounds, thereby increasing their fitness in different environments [30].

### Functional diversity of microbiota in the earthworm gut

We found that cave earthworms harbor more diverse functional genes in their gut, despite lower taxonomic and phylogenetic diversities, supporting our first hypothesis. Both earthworms enriched the genes related to the function of metabolism in their gut microbiomes, which is likely to provide essential metabolites to them and increase their survival rates [31]. Higher proportions of metabolism-related genes were found in the gut of soil animal microbiota compared to other functions. It is evidenced soil animals are highly dependent on the metabolism genes from their gut microbiome for nutrient needs [32, 33]. As the cave environment is deficient in food sources, when the earthworms are living in the caves, their gut might be stimulated to serve as a more efficient “nutrient factory” [12]. Notably, a large proportion of the genes, especially from the gut microbiome of earthworms in the caves, was unclassified. This demonstrates that the gut microbiome of cave earthworms is more functionally diverse than has been seen. More studies integrating new technologies should be conducted to uncover their roles.

### Deterministic community assembly in the earthworm gut

Our results show that the assembly processes of earthworm gut microbiome were deterministic, supporting our second hypothesis. The oxygen and water contents differed along the digestive tract of earthworms, thus the profile of microbiota is shaped by the digestive environment [34–36]. In addition, studies have revealed that the food source is another deterministic factor shaping the gut microbiome [37–40], both phylogeny and food preference of the host may deterministically shape the gut microbiome of soil animals [41]. For surviving in caves, earthworms therefore might select microbiota coding more metabolic activities.

### Community features of gut microbiome

The fact that gut microbiome holds greater numbers of CWM copy numbers of the 16S rRNA gene than soil microbiota suggesting a more nutrient demand in the gut microbiome. The multiplicity of rRNA genes is an indicator of the ecological strategy of bacteria for nutrient exploitation [18]. For example, during the exponential growth phase, the number of rRNA operons of *Escherichia coli* may increase from 7 to 36 [42, 43]. Therefore, communities dominated by bacteria with fewer *rrn* copy numbers usually have a higher nutrient use efficiency than high-*rrn*-dominated communities [44]. In caves, where soil organic matter is different compared with surface systems, earthworms might benefit from the help of a highly efficient gut microbiome supporting higher metabolism [45]. That was the reason *A. chlorotica* established a more stable population, compared with *A. rosea*.

## Conclusions

The present study demonstrates the functional roles of gut microbiome in contributing to host adaptations of two earthworms in surface and cave environments. Our results reflect the tight interactions between host earthworms and their gut microbiome. The gut microbiome exhibits a more functional diversity in caves, which can be interpreted as evidence of stronger food limitation. A more stable and highly efficient microbiota providing metabolites is needed for the earthworms to survive in the resource-limited cave habitat. Together, the gut microbiome-host crosstalk is of pivotal importance in facilitating the animal hosts in their physiological adaptation and even the population expansion.

## Supplementary Information

**Additional file 1. Figure S1 **The diversity of predicted functional genes in soil and gut ofearthworms present in caves and the surface. Functions were predicted using PICRUSt.

## Data Availability

The sequences are available at the NCBI Sequence Read Archive under the accession numbers PRJNA400302.

## References

[1] Poulson TL, White WB (1969). The cave environment. Science.

[2] Christman MC, Culver DC, Madden MK, White D (2005). Patterns of endemism of the eastern North American cave fauna. J Biogeogr.

[3] Christman MC, Culver DC (2001). The relationship between cave biodiversity and available habitat. J Biogeogr.

[4] Mammola S (2019). Finding answers in the dark: caves as models in ecology fifty years after Poulson and White. Ecography (Cop).

[5] Smrž J, Kováč L, Mikeš J, Šustr V, Lukešová A, Tajovský K (2015). Food sources of selected terrestrial cave arthropods. Subterr Biol.

[6] Phillips HRP, Guerra CA, Bartz MLC, Briones MJI, Brown G, Crowther TW (2019). Global distribution of earthworm diversity. Science.

[7] Reeves WK, Reynolds JW (1999). New records of cave-dwelling earthworms (Oligochaeta: Lumbricidae, Megascolecidae and Naididae) and other annelids (Aeolosomatida, Branchiobdellida and Hirudinea) in the Southeastern United States, with notes on their ecology. Megadrilogica.

[8] Reynolds JW. Note on some cave earthworms (Oligochaeta: Lumbricidae) from the Isle of Man, U.K. Megadrilogica. 1996;6:89–90.

[9] Protas ME, Trontelj P, Patel NH (2011). Genetic basis of eye and pigment loss in the cave crustacean, *Asellus aquaticus*. Proc Natl Acad Sci U S A.

[10] Ley RE, Lozupone CA, Hamady M, Knight R, Gordon JI (2008). Worlds within worlds: evolution of the vertebrate gut microbiota. Nat Rev Microbiol.

[11] Shapira M (2016). Gut microbiotas and host evolution: scaling up symbiosis. Trends Ecol Evol.

[12] Ankrah NYD, Douglas AE (2018). Nutrient factories: metabolic function of beneficial microorganisms associated with insects. Environ Microbiol.

[13] Chu CC, Spencer JL, Curzi MJ, Zavala JA, Seufferheld MJ (2013). Gut bacteria facilitate adaptation to crop rotation in the western corn rootworm. Proc Natl Acad Sci U S A.

[14] Yun JH, Roh SW, Whon TW, Jung MJ, Kim MS, Park DS (2014). Insect gut bacterial diversity determined by environmental habitat, diet, developmental stage, and phylogeny of host. Appl Environ Microbiol.

[15] Thakuria D, Schmidt O, Finan D, Egan D, Doohan FM (2010). Gut wall bacteria of earthworms: a natural selection process. ISME J.

[16] Rossmassler K, Dietrich C, Thompson C, Mikaelyan A, Nonoh JO, Scheffrahn RH, et al. Metagenomic analysis of the microbiota in the highly compartmented hindguts of six wood- or soil-feeding higher termites. Microbiome. 2015;:1–6. 10.1186/s40168-015-0118-1 PMC4660790 26607965

[17] Ding J, Zhu D, Li H, Ding K, Chen QL, Lassen SB (2019). The gut microbiota of soil organisms show species-specific responses to liming. Sci Total Environ.

[18] Klappenbach JA, Dunbar JM, Schmidt TM (2000). rRNA operon copy number reflects ecological strategies of bacteria. Appl Environ Microbiol.

[19] Layeghifard M, Hwang DM, Guttman DS (2017). Disentangling interactions in the microbiome: a network perspective. Trends Microbiol.

[20] Růžička V, Šmilauer P, Mlejnek R (2013). Colonization of subterranean habitats by spiders in Central Europe. Int J Speleol.

[21] Rutgers M, Orgiazzi A, Gardi C, Römbke J, Jänsch S, Keith AM (2016). Mapping earthworm communities in Europe. Appl Soil Ecol.

[22] Caporaso JG, Kuczynski J, Stombaugh J, Bittinger K, Bushman FD, Costello EK (2010). QIIME allows analysis of high-throughput community sequencing data. Nat Methods.

[23] Langille MGI, Zaneveld J, Caporaso JG, McDonald D, Knights D, Reyes JA (2013). Predictive functional profiling of microbial communities using 16S rRNA marker gene sequences. Nat Biotechnol.

[24] R Core Team. R: a language and environment for statistical computing. R foundation for statistical computing, Vienna, Austria. URL http://www.R-project.org/. 2021.

[25] Kembel SW, Cowan PD, Helmus MR, Cornwell WK, Morlon H, Ackerly DD (2010). Picante: R tools for integrating phylogenies and ecology. Bioinformatics.

[26] Schloss PD, Westcott SL, Ryabin T, Hall JR, Hartmann M, Hollister EB (2009). Introducing mothur: open-source, platform-independent, community-supported software for describing and comparing microbial communities. Appl Environ Microbiol.

[27] Csardi G, Nepusz T. The igraph software package for complex network research. InterJournal. 2006; Complex Systems:1695.

[28] Bredon M, Dittmer J, Noël C, Moumen B, Bouchon D (2018). Lignocellulose degradation at the holobiont level: teamwork in a keystone soil invertebrate. Microbiome.

[29] O’brien PA, Webster NS, Miller DJ, Bourne DG. Host-microbe coevolution: Applying evidence from model systems to complex marine invertebrate holobionts. MBio. 2019;10:1–14. 10.1128/mBio.02241-18 PMC6428750 30723123

[30] Macke E, Tasiemski A, Massol F, Callens M, Decaestecker E (2017). Life history and eco-evolutionary dynamics in light of the gut microbiota. Oikos.

[31] Bon D, Gilard V, Massou S, Pérès G, Malet-Martino M, Martino R (2006). In vivo ^31^P and ^1^H HR-MAS NMR spectroscopy analysis of the unstarved *Aporrectodea caliginosa* (Lumbricidae). Biol Fertil Soils.

[32] Xiang Q, Zhu D, Chen QL, Delgado-Baquerizo M, Su JQ, Qiao M (2019). Effects of diet on gut microbiota of soil collembolans. Sci Total Environ.

[33] Mathipi V, de Mandal S, Chawngthu Z, Lalfelpuii R, Kumar NS, Lalthanzara H (2020). Diversity and metabolic potential of earthworm gut microbiota in Indo-Myanmar biodiversity hotspot. J Pure Appl Microbiol.

[34] Sampedro L, Whalen JK (2007). Changes in the fatty acid profiles through the digestive tract of the earthworm Lumbricus terrestris L. Appl Soil Ecol.

[35] Drake HL, Horn MA (2007). As the Worm Turns: The earthworm gut as a transient habitat for soil microbial biomes. Annu Rev Microbiol.

[36] Trigo D, Lavelle P (1993). Changes in respiration rate and some physicochemical properties of soil during gut transit through *Allolobophora molleri* (Lumbricidae, Oligochaeta). Biol Fertil Soils.

[37] Knapp BA, Seeber J, Podmirseg SM, Meyer E, Insam H (2008). Application of denaturing gradient gel electrophoresis for analysing the gut microflora of Lumbricus rubellus Hoffmeister under different feeding conditions. Bull Entomol Res.

[38] Knapp BA, Podmirseg SM, Seeber J, Meyer E, Insam H (2009). Diet-related composition of the gut microbiota of *Lumbricus rubellus* as revealed by a molecular fingerprinting technique and cloning. Soil Biol Biochem.

[39] Rudi K, Ødegård K, Løkken TT, Wilson R (2009). A feeding induced switch from a variable to a homogenous state of the earthworm gut microbiota within a host population. PLoS ONE.

[40] Egert M, Marhan S, Wagner B, Scheu S, Friedrich MW. Molecular profiling of 16S rRNA genes reveals diet-related differences of microbial communities in soil, gut, and casts of Lumbricus terrestris L. (Oligochaeta: Lumbricidae). FEMS Microbiol Ecol. 2004;48:187–97. 10.1016/j.femsec.2004.01.007 19712402

[41] Gong X, Chen TW, Zieger SL, Bluhm C, Heidemann K, Schaefer I (2018). Phylogenetic and trophic determinants of gut microbiota in soil oribatid mites. Soil Biol Biochem.

[42] Condon C, Liveris D, Squires C, Schwartz I, Squires CL (1995). rRNA operon multiplicity in *Escherichia coli* and the physiological implications of rrn inactivation. J Bacteriol.

[43] Klappenbach JA, Saxman PR, Cole JR, Schmidt TM (2001). rrndb: The ribosomal RNA operon copy number database. Nucleic Acids Res.

[44] Roller BRK, Stoddard SF, Schmidt TM. Exploiting rRNA operon copy number to investigate bacterial reproductive strategies. Nat Microbiol. 2016;1 November:1–7. 10.1038/nmicrobiol.2016.160 PMC5061577 27617693

[45] Valdivia-Anistro JA, Eguiarte-Fruns LE, Delgado-Sapién G, Márquez-Zacarías P, Gasca-Pineda J, Learned J (2016). Variability of rRNA operon copy number and growth rate dynamics of bacillus isolated from an extremely oligotrophic aquatic ecosystem. Front Microbiol.

