# Supplementary figures and images for: Gut microbiome reflect adaptation of earthworms to cave and surface environments
Source: Anim Microbiome. 2022 Aug 5;4:47. doi: 10.1186/s42523-022-00200-0 (PMC9356433; doi:10.1186/s42523-022-00200-0)

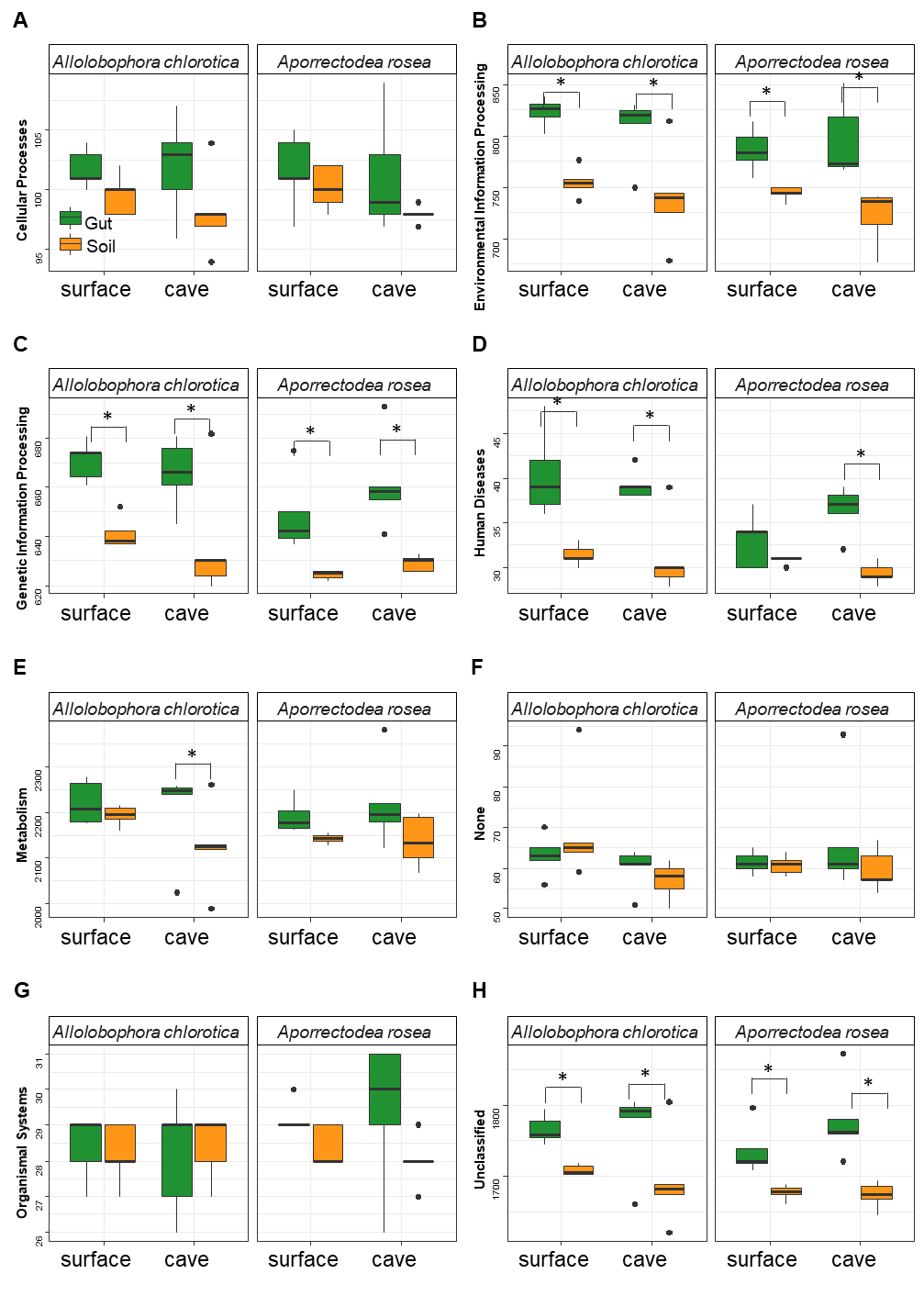

Supplement: Supplementary file 1 — Additional file 1. Figure S1 The diversity of predicted functional genes in soil and gut ofearthworms present in caves and the surface. Functions were predicted using PICRUSt. [file 42523_2022_200_MOESM1_ESM.png]
